# Recombinant Human Acid Sphingomyelinase as an Adjuvant to Sorafenib Treatment of Experimental Liver Cancer

**DOI:** 10.1371/journal.pone.0065620

**Published:** 2013-05-28

**Authors:** Radoslav Savić, Xingxuan He, Isabel Fiel, Edward H. Schuchman

**Affiliations:** 1 Department of Genetics and Genomic Sciences, Icahn School of Medicine at Mount Sinai, New York, New York, United States of America; 2 Department of Pathology, Icahn School of Medicine at Mount Sinai, New York, New York, United States of America; Christian-Albrechts-University Kiel, Germany

## Abstract

**Background:**

Hepatocellular carcinoma (HCC) is the most common form of liver cancer and the third leading cause of cancer death worldwide. The only approved systemic treatment for unresectable HCC is the oral kinase inhibitor, sorafenib. Recombinant human acid sphingomyelinase (rhASM), which hydrolyzes sphingomyelin to ceramide, is an orphan drug under development for the treatment of Type B Niemann-Pick disease (NPD). Due to the hepatotropic nature of rhASM and its ability to generate pro-apoptotic ceramide, this study evaluated the use of rhASM as an adjuvant treatment with sorafenib in experimental models of HCC.

**Methodology/Principal Findings:**

*In vitro*, rhASM/sorafenib treatment reduced the viability of Huh7 liver cancer cells more than sorafenib. *In vivo*, using a subcutaneous Huh7 tumor model, mouse survival was increased and proliferation in the tumors decreased to a similar extent in both sorafenib and rhASM/sorafenib treatment groups. However, combined rhASM/sorafenib treatment significantly lowered tumor volume, increased tumor necrosis, and decreased tumor blood vessel density compared to sorafenib. These results were obtained despite poor delivery of rhASM to the tumors. A second (orthotopic) model of Huh7 tumors also was established, but modest ASM activity was similarly detected in these tumors compared to healthy mouse livers. Importantly, no chronic liver toxicity or weight loss was observed from rhASM therapy in either model.

**Conclusions/Significance:**

The rhASM/sorafenib combination exhibited a synergistic effect on reducing the tumor volume and blood vessel density in Huh7 xenografts, despite modest activity of rhASM in these tumors. No significant increases in survival were observed from the rhASM/sorafenib treatment. The poor delivery of rhASM to Huh7 tumors may be due, at least in part, to low expression of mannose receptors. The safety and efficacy of this approach, together with the novel findings regarding enzyme targeting, merits further investigation.

## Introduction

The most recent estimate by the American Cancer Society for 2013 is that about 30,640 people would be diagnosed with primary liver and bile duct cancer in the United States, with about 21,670 (71%) cancer related deaths. HCC is the most common (∼90%) form of liver cancer, often diagnosed at advanced stages of the disease [1]. HCC is a genetically heterogeneous malignancy in which numerous deregulated signaling pathways lead to elevated proliferation and angiogenesis, including RAF/MEK/ERK, PI3K/AKT/mTOR, WNT/β-catenin, IGF, and HGF/c-MET [2]. Until recently, treatment options for advanced/unresectable HCC have been relatively ineffective and complicated by the underlying hepatitis and liver cirrhosis. In 2007 the FDA approved an oral drug for unresectable HCC – sorafenib, a small molecule multikinase inhibitor with an *in vitro* activity against dozens of serine/threonine (e.g., RAF) and tyrosine kinases (e.g., VEGFR) in tumor cells and vasculature [3], [4]. In pivotal clinical studies, sorafenib afforded 2.8 months better survival in the treatment group (10.7 months median) compared to placebo (7.9 months), forming the basis of its approval by the FDA [3], [5]. However, despite the demonstrated clinical efficacy, some patients with advanced disease fail to respond to sorafenib and those that do have a finite benefit [5]. Consequently, investigations into alternative/supportive drug treatments have been gaining momentum [6].

In contrast to HCC, NPD comprises a family of ultra rare monogenic disorders with known genetic and biochemical abnormalities. For example, mutations in the *SMPD1* gene result in the deficiency of ASM activity, leading to accumulation of sphingomyelin in lysosomes and other cellular compartments. Type A NPD is the neurodegenerative, infantile form of ASM deficiency, usually fatal within the first 2–3 years of life. In contrast, Type B NPD lacks neurological involvement and survival may be into late childhood or adulthood, although affected individuals frequently exhibit progressive hepatosplenomegaly and respiratory illness [7]. Enzyme replacement therapy with exogenous rhASM received orphan drug status for Type B NPD in 2000 [8], and has been successfully tested in a phase I clinical trial in adult Type B NPD patients (clinicaltrials.gov identifier NCT00410566). A phase Ib repeat dosing study is underway. The hydrolysis of sphingomyelin by rhASM produces a highly bioactive and cytocidal lipid, ceramide, which is capable of inducing tumor suppression [9]. It is known that elevation of ceramide at the cell surface re-organizes cell membrane signaling platforms, likely inducing the downstream cellular changes, but the exact mechanisms underlying these effects remains an active area of investigation [9].

Due to the pro-death effects of ceramide, cancer cells have developed multiple defense mechanisms to overcome this lipid, including reduced production and/or enhanced clearance, or elevated production of the counteracting pro-survival lipid, sphingosine-1-phosphate (S1P). These defense mechanisms also may contribute to sphingolipid-mediated drug resistance [10], [11]. Consequently, drug therapies targeting sphingolipid metabolism, including overproduction of ceramide to kill tumor cells or reduce angiogenesis, represent attractive approaches for cancer treatment. Many of these new sphingolipid drug therapies have been evaluated in cell culture and/or animal models, and are focused on direct distribution of non-physiological ceramides [12] to tumors, or administration of inhibitors of ceramidases or the sphingosine kinases responsible for the synthesis of S1P [13]. Since rhASM is a) selectively taken up by the liver after systemic administration, b) highly effective in generating ceramide, and c) available in a clinical grade formulation, we focused on investigating the potential of rhASM as an adjuvant to sorafenib treatment in experimental liver cancer.

Previously, we showed that rhASM in combination with irradiation had a profound effect on melanoma *in vivo.* To recapitulate this effect *in vitro* the media needed to be acidified (pH 6.5), a condition that mimics the microenvironment of the tumor and favors ASM activity [14]. We also showed that rhASM alone (1 µM) had no reproducible effect on the viability of 60 cancer cell lines encompassing leukemia, non-small cell lung, colon, CNS, melanoma, ovarian, renal, prostate and breast tumors, suggesting that the *in vivo* microenvironment of the tumor was important for the observed effects [15], [16].

Considering the hepatotropic nature of rhASM, we hypothesized that liver cancer may be an appropriate model in which to next test the efficacy of rhASM as an adjuvant to standard of care sorafenib treatment. Here, we demonstrate that combination treatment with high dose rhASM (25 mg/kg every three days (q.72 h), intraperitoneally (i.p.)) and sorafenib (30 mg/kg every day (q.d.), gavage) reduces tumor volume of Huh7 subcutaneous xenografts *in vivo*, reduces blood vessel density, and results in increased necrosis in the tumors. These effects were obtained despite limited delivery of the enzyme to the subcutaneous tumors. The combination treatment was well tolerated in BALB/C mice without any treatment related deaths, without loss of weight, and with normal liver function. We also established an orthotopic model of Huh7 tumors in livers of SCID/beige mice, and surprisingly found similarly poor delivery of rhASM to these tumors relative to healthy liver. Further investigation suggested that low expression of mannose receptors in Huh7 tumors might partially explain this effect.

## Results

### Deregulation of sphingolipid signaling in human hepatocellular carcinoma

**Figure 1:**
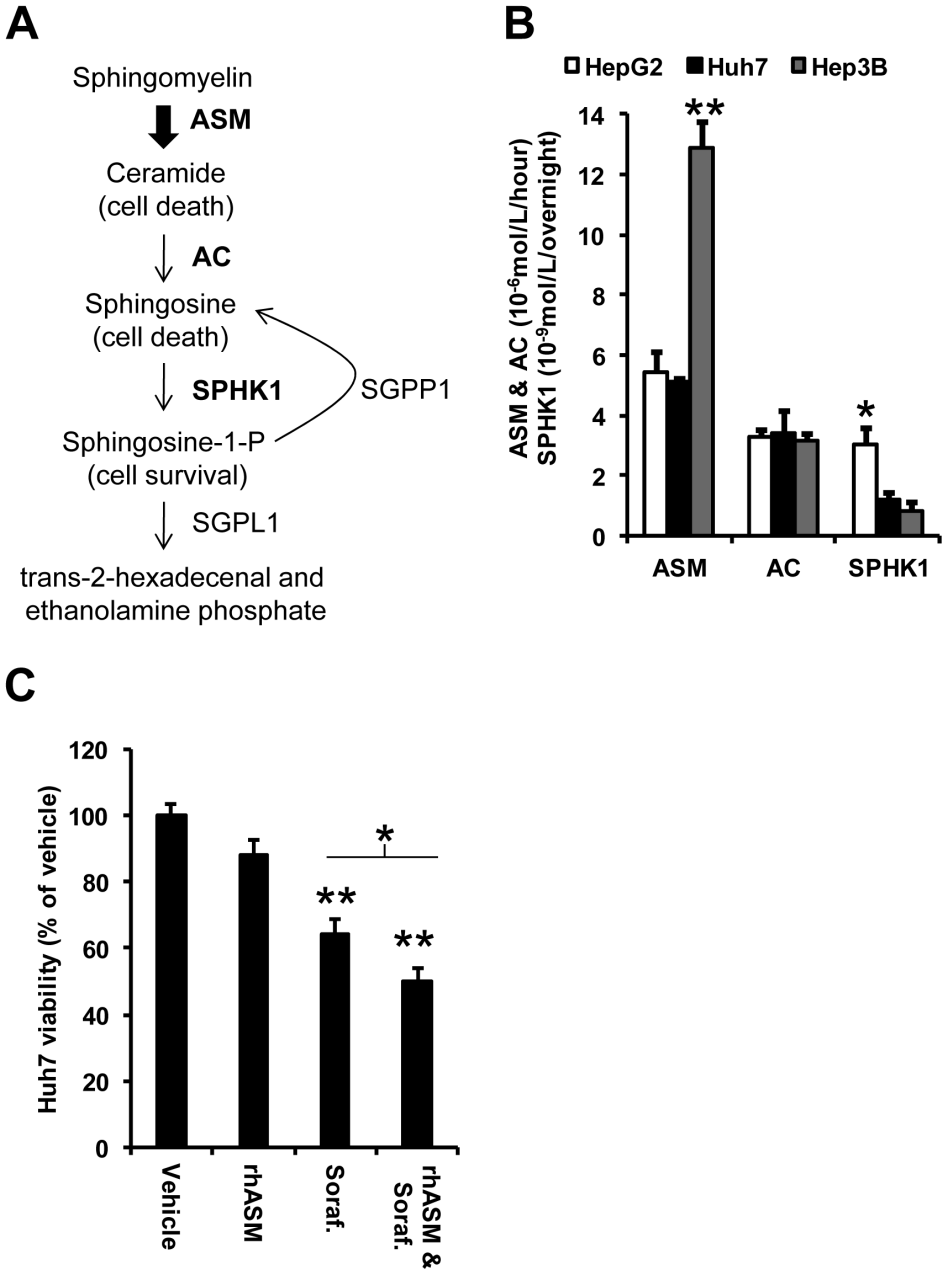
Rationale and selection of human hepatoma cells. (A) ASM drives the production of pro-apoptotic ceramide through the hydrolysis of sphingomyelin, which is converted to sphingosine by ceramidases such as acid ceramidase (AC). Sphingosine kinase 1 (SPHK1) then phosphorylates sphingosine to sphingosine-1-phosphate (S1P), which is converted back to sphingosine by S1P-phosphatase (SGPP1) or metabolized by S1P lyase 1 (SGPL1). (B) Activity of ASM in Hep3B cells was significantly higher (ANOVA, df (2,6), F = 48.49, p<0.001) than in Huh7 (Tukey's post hoc test p<0.001, **) and HepG2 cells (Tukey's post hoc test p<0.001). AC was similar across all cell lines, but HepG2 cells had significantly higher SPHK1 activity (ANOVA, df (2,6), F = 8.68, p = 0.017, *) than Huh7 (Tukey's post hoc test, p = 0.041) and Hep3B (Tukey's post hoc test, p = 0.019). (C) Huh7 cells were selected for further studies and their viability tested at pH 6.5 (see Methods) in the presence of 500 µg/mL rhASM, 3 µM sorafenib, or the combination of rhASM and sorafenib at 48 hours. Sorafenib (Dunnett's post hoc test p<0.001, **) and combined rhASM/sorafenib (Dunnett's post hoc test p<0.001, **) treated cells had significantly lower viability than control cells (ANOVA, df (3,38), F = 26.47, p<0.001). rhASM was not significantly different from control (p = 0.118). The rhASM and sorafenib combination exhibited significantly lower viability compared to sorafenib alone (t-test, 1-sided, *p<0.05, **p<0.001).

Comparison of normal livers to hepatocellular carcinomas, using four independent sets of samples available in the Oncomine database, revealed significantly decreased levels of ASM (*SMPD1*) and S1P phosphatase (*SGPP1*) mRNA expression (Table 1). The *SMPD1* gene ranked among the top 1, 9, 17 and 11% of repressed genes in the Mas [17], Chen [18], Wurmbach [19], and Roessler liver 2 datasets [20]. Similarly, the *SGPP1* gene was ranked in the top 4, 5 and 7% repressed genes in 3 out of 4 datasets [17], [18], [19]. S1P is a highly bioactive sphingolipid that promotes cell proliferation [11], and S1P phosphatase is the enzyme required to hydrolyze the phosphate group from S1P (Figure 1A). Repression of these two genes/enzymes would therefore favor low ceramide and high S1P levels, likely leading to cell proliferation and/or drug resistance.

**Table 1:** Decreased expression of *SMPD1* and *SGPP1* genes in HCC.

Gene symbol:	*SMPD1*	*SGPP1*	*SMPD1*	*SGPP1*	*SMPD1*	*SGPP1*	*SMPD1*	*SGPP1*
Oncomine set:	Mas	liver	Chen	liver	Wurmbach	liver	Roessler	Liver2
Liver samples:	19		76		10		220	
HCC samples:	38		103	104	35		225	
Genes analyzed:	12603		10802		19574		12624	
Fold change:	−2.144	−1.961	−1.429	−1.631	−1.716	−2.460	−1.373	−1.075
T-test:	−9.045	−6.746	−5.780	−7.602	−2.758	−4.045	−8.498	−1.649
P value:	1.2E-12	1.6E-08	1.7E-08	1.1E-12	9.0E-03	5.6E-04	2.2E-16	0.050
Gene rank:	33	388	942	500	3320	1203	1329	5991
Gene rank %:	Top 1%	Top 4%	Top 9%	Top 5%	Top 17%	Top 7%	Top 11%	Top 48%
mRNA in HCC:	↓	↓	↓	↓	↓	↓	↓	↓

Significantly lower mRNA expression levels of the *SMPD1* and *SGPP1* genes were found in HCC samples compared to normal livers (↓ under-expressed). Four human data sets were accessed using the Oncomine database: Mas liver [17], Chen Liver [18], Wurmbach liver [19], and Roessler Liver 2 [20].

### Selection of a human hepatoma cell line and *in vitro* effect of rhASM on proliferation

We next investigated the baseline activities of three key enzymes involved in sphingolipid metabolism – ASM, acid ceramidase (AC), and sphingosine kinase 1 (SPHK1) – in three commonly used human hepatoma cell lines: HepG2, Huh7 and Hep3B. Hep3B cells had the highest baseline ASM activity, while SPHK1 activity was highest in HepG2 cells (Figure 1B). All three cells had comparable AC activity. Based on these results we selected Huh7 cells for further experiments since the baseline activities were moderate and between those of HepG2 and Hep3B. In addition, Huh7 subcutaneous mouse xenografts are a well-established model used to assess diverse experimental drug treatments for HCC [21], [22], [23]. The effect of rhASM was investigated in Huh7 cells by pre-treatment (2 hours) with the rhASM in an acidified medium (6.5) – mimicking the tumor microenvironment pH – followed by incubation for 46 hours at pH 7.4. No significant effect of rhASM treatment alone was observed on the viability of Huh7 cells during the 48 hours, as was observed previously in melanoma cells [14]. In contrast, sorafenib led to a significant reduction in proliferation, which was significantly enhanced in cells exposed to rhASM/sorafenib combination (Figure 1C). These data further supported the notion that rhASM alone has little effect on tumor cells, and may be useful as an adjuvant treatment to sorafenib [14].

### Reduced tumor volume and increased survival in mice treated with rhASM and sorafenib

**Figure 2:**
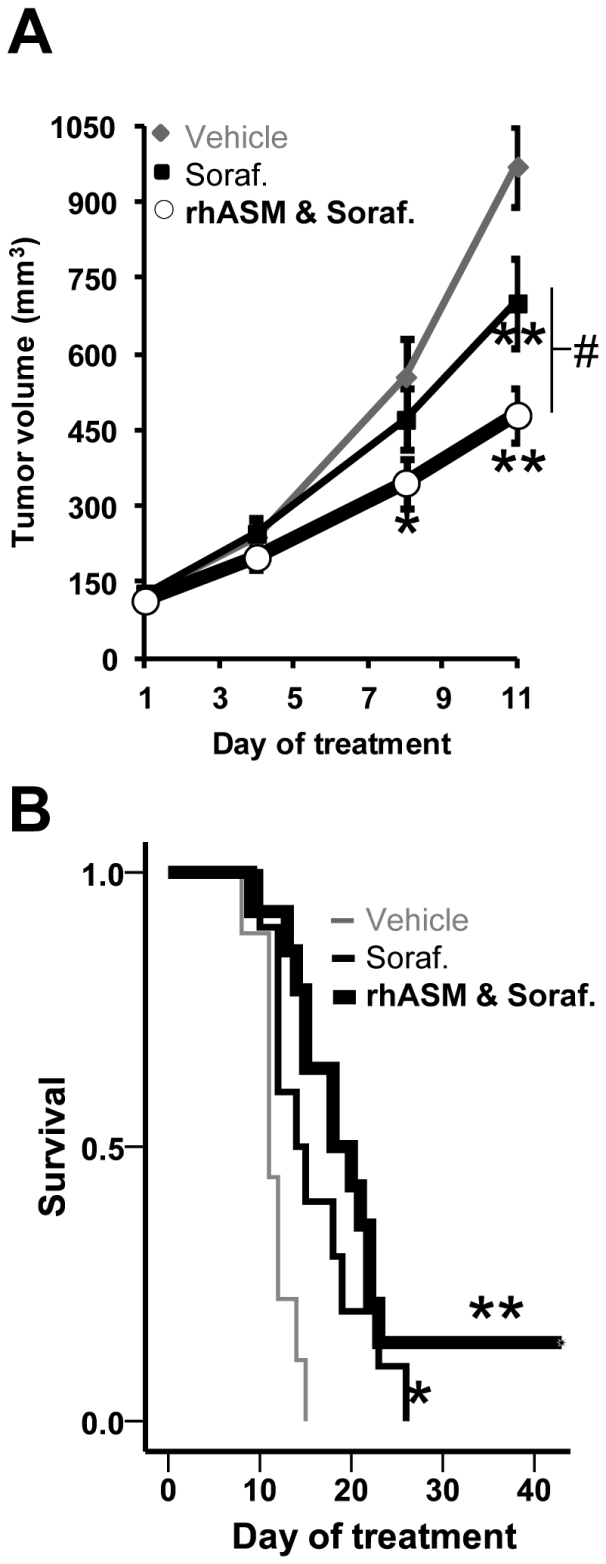
rhASM/sorafenib co-treatment reduces tumor volume and shows a trend towards improved survival in mice bearing subcutaneous Huh7 xenografts versus sorafenib alone. (A) The mean tumor volume of mice treated with rhASM and sorafenib was significantly smaller than that of control mice at day 8 (Dunnett's post-hoc test p = 0.035; ANOVA df (2,30), F = 3.24, p = 0.053). At day 11 both sorafenib (Dunnett's post hoc test p = 0.034) and combined rhASM and sorafenib (Dunnett's post hoc test p<0.001) treated mice had smaller tumors than control mice (ANOVA, df (2,27), F = 12.22, p<0.001). The rhASM/sorafenib combination group also had significantly smaller tumors than the sorafenib group at day 11 (t = 2.32, df (20), p = 0.031). (B) Significantly longer median survival (13 days) of sorafenib treated mice (chi-square 5.02, df (1), p = 0.025) and combined rhASM/sorafenib treated mice (19 days) (chi-square 14.57, df (1), p<0.001) was observed compared to control (11 days). Two mice in the rhASM/sorafenib group lived beyond the 5 week study period, and were eventually sacrificed at day 43 (tumor volume 132 mm^3^, 267 mm^3^). *, # p<0.05, ** p<0.001.

Next, we investigated the effects of rhASM/sorafenib combination treatment *in vivo* using Huh7 subcutaneous xenograft tumors in mice. Animals were randomized into 4 groups treated with sorafenib (n = 10), rhASM (n = 13), rhASM/sorafenib (n = 14), or vehicle (n = 9). As in the *in vitro* experiments, rhASM treatment alone had no beneficial effect *in vivo* (median survival 10 days, tumor volume 925±80 mm^3^ on day 11) (data not shown). However, compared to vehicle, significantly lower tumor volumes were measured in the rhASM/sorafenib group at days 8 and 11. Also, at day 11 the reduction of tumor volume observed in the combination group was significantly lower than in mice treated with sorafenib alone (Figure 2A). The survival of mice was significantly greater in both the sorafenib (13 days) and combination treated mice (19 days) compared to vehicle (11 days) (Figure 2B). Although there was a trend towards improvement, no significant survival differences were observed between the two treatment groups. Of note, however, two animals in the rhASM/sorafenib combination group had tumors <1,000 mm^3^ and survived beyond the 5 weeks. These mice were sacrificed at day 43 as their tumor volumes remained relatively stable (132 mm^3^ (mouse ID#452) and 267 mm^3^ (mouse ID#443) at the time of sacrifice.

### Reduced proliferation and blood vessel density and increased cell death in tumors from combined rhASM/sorafenib treated mice

**Figure 3:**
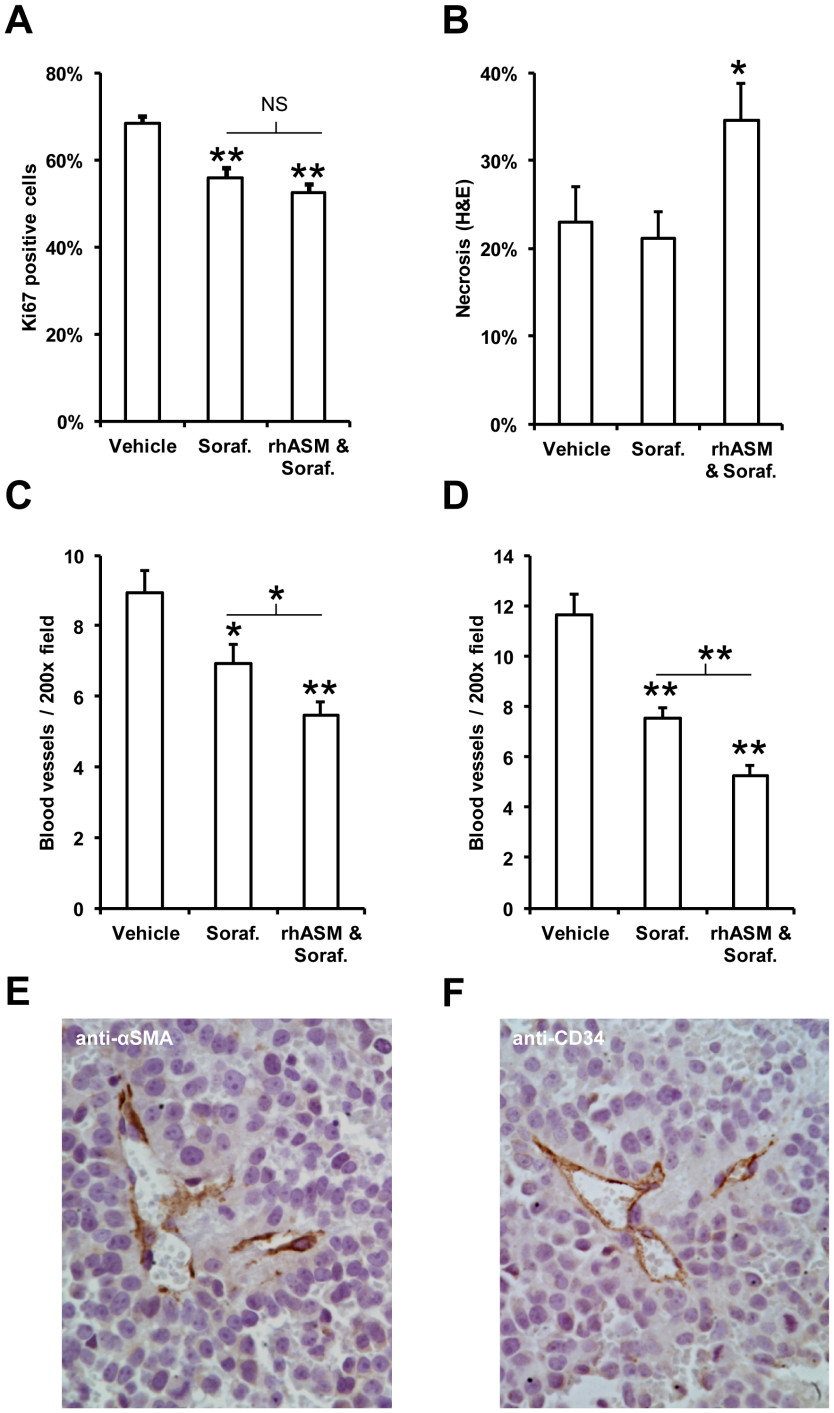
rhASM/sorafenib co-treatment reduces blood vessel density and increases necrosis in Huh7 tumors. (A) Mean number of Ki67 positive cells in tumors from mice treated with sorafenib (Dunnett's post-hoc p<0.005) and with rhASM/sorafenib combination (Dunnett's post-hoc p<0.001) was significantly lower than vehicle (ANOVA df (2,30), F = 14.63, p<0.001). No significant difference was observed between Ki67 staining in tumors from sorafenib and rhASM/sorafenib treated mice (t = 1.19, df 22, p = 0.249). (B) Necrosis was significantly increased (ANOVA, df (2,30), F = 3.66, p = 0.038) in rhASM/sorafenib treated mice (Dunnett's post hoc > control, p = 0.043) compared to vehicle treated mice, while sorafenib treated mice were not different from control (Dunnett's post hoc > control, p = 0.760). Necrosis in rhASM/sorafenib treated mice was significantly greater than in mice treated with sorafenib alone (t = −2.39, df (22), p = 0.26). (C) Anti-αSMA blood vessel staining revealed a significantly lower number of vessels (ANOVA df (2,30), F = 12.57, p<0.005) in sorafenib (Dunnett's post hoc test p = 0.020) and rhASM/sorafenib treated mice (Dunnett's post hoc test p<0.001). The rhASM/sorafenib group also had significantly less αSMA blood vessels than the sorafenib group (t = 2.25, df (22), p = 0.031). (D) Anti-CD34 blood vessel staining confirmed the αSMA results, showing a significantly lower number of vessels (ANOVA df (2,30), F = 32.07, p<0.001) in sorafenib (Dunnett's post hoc test p<0.001) and rhASM/sorafenib treated mice (Dunnett's post hoc test p<0.001). The combined rhASM/sorafenib group had significantly less CD-34 positive blood vessels than the sorafenib group (t = 3.56, df (22), p = 0.002). Selective staining of blood vessels in paraffin embedded tumor sections stained is shown with anti- αSMA (E) and anti-CD34 markers (F). *p<0.05, **p<0.001.

At the molecular level, the number of cells positive for the Ki67 proliferation marker was significantly decreased in both the sorafenib and rhASM/sorafenib treatment groups to a similar extent (Figure 3A). However, necrosis was significantly increased in the combined rhASM/sorafenib treated mice (Figure 3B). To investigate this finding further, we next examined vascularization of the tumors. The number of blood vessels stained with anti-αSMA was significantly lower in tumors from both sorafenib (6.9±0.5) and rhASM/sorafenib (5.5±0.4) treated mice compared to control (9±0.6). Importantly, the number of anti-αSMA positive blood vessels was significantly lower in rhASM/sorafenib than in sorafenib treated mice (p<0.001). Similar results were obtained by anti-CD34 staining, where rhASM/sorafenib (5.3±0.4) was significantly lower than sorafenib alone (7.5±0.4), and both were lower than control (11.6±0.9). Both anti-αSMA and anti-CD34 allowed for selective staining of blood vessels in paraffin embedded tumor sections, as depicted in Figures 3 E,F. The rhASM/sorafenib long-surviving mice (ID#452 and ID#443) were within the range of measurements for the combination group using either of the assays above.

The analysis of ceramide levels in tumors, which showed no difference between the groups (data not shown), was done as an endpoint measurement at the completion of the study (up to 48 hours after the last injection). Since the elevation of ceramide in cells in response to ASM is rapid and may return to baseline within minutes, we looked at tumor necrosis and blood vessel density (above) as surrogate markers for the biological effects of treatment. Since we observed a decrease in tumor volume, increase in necrosis, and decrease in blood vessel density in the rhASM/sorafenib group, we did not measure the levels of other sphingolipid metabolites such as S1P. In general, however, it is clear from our data that the predominant effect of rhASM combination treatment was cell death, and thus any downstream S1P that may have been generated did not prevent these rhASM/sorafenib-induced changes.

### Modest distribution of rhASM into subcutaneous tumors compared to healthy liver

**Figure 4:**
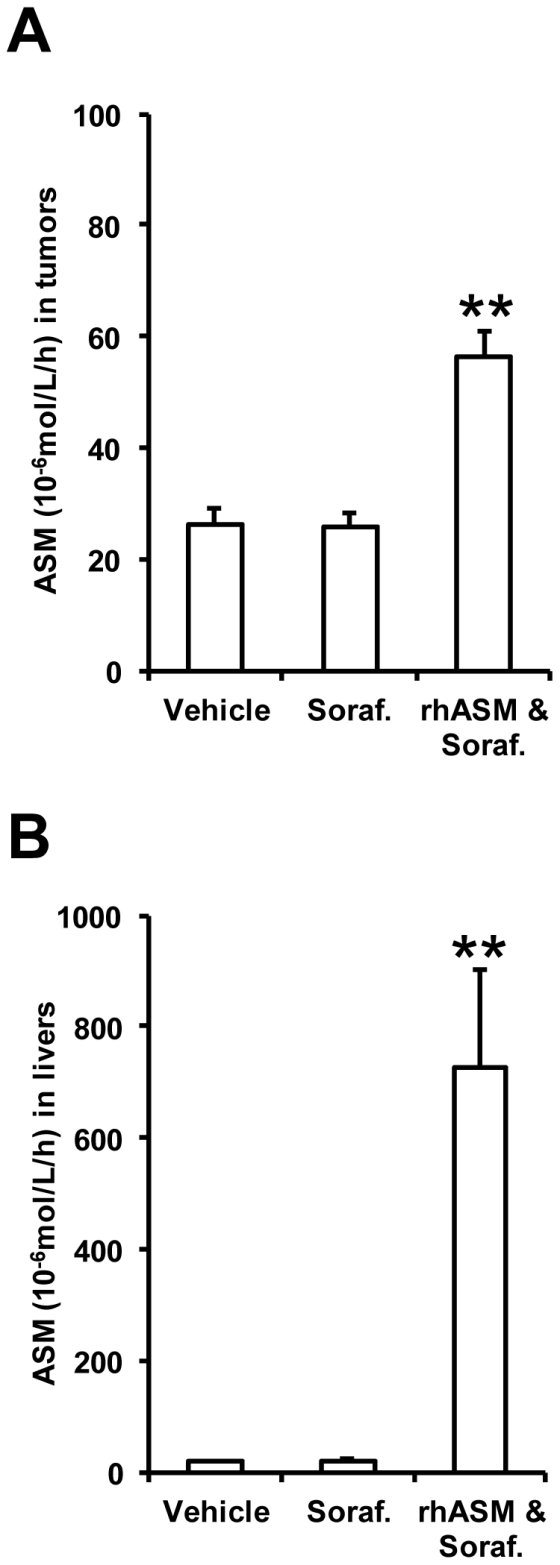
Localization of rhASM in subcutaneous tumors and livers. (A) ASM activity in tumor extracts of rhASM/sorafenib treated mice was significantly higher (ANOVA, df (2,30), F = 22.58, p<0.001; Dunnett's post hoc test p<0.001) than control, while sorafenib had no effect on baseline ASM activity. (B) ASM activity in liver extracts of rhASM/sorafenib treated mice also was higher than vehicle treated mice (ANOVA, df (2,30), F = 11.17, p<0.001; Dunnett's post hoc test p<0.001), and sorafenib had no effect on baseline ASM activity. Of note, animals in the combination treatment group had >12 times higher ASM activity in livers than in tumors, demonstrating the hepatotropic nature of rhASM during chronic administration and relatively modest distribution to the subcutaneous tumors. ** p<0.001. Note: Y-axes activity (10^−6^ mol/L/hour) was measured in equal parts of 20% weight/volume tissue extracts as described in Materials and Methods.

The above data demonstrated a positive, although modest, impact of combined rhASM/sorafenib treatment in this subcutaneous Huh7 HCC model. To examine the biodistribution of the enzyme to the tumors, we measured ASM activity at the end of the study. Each animal received the last rhASM injection 1, 24 or 48 hours prior to sacrifice according to the individual animal's dosing regimen. No apparent differences in ASM activity were observed in relation to the time waited after the last rhASM injection. As expected, the ASM activity was significantly higher in tumor extracts from the rhASM/sorafenib treated mice compared to sorafenib alone (Figure 4A). However, ASM activity in the healthy livers of these mice was >12 times that in the subcutaneous tumors (Figure 4B). Thus, modest biodistribution of rhASM to the subcutaneous tumors may explain the modest efficacy of the combination treatment in this model.

### Safety of rhASM/sorafenib combination treatment

In the phase I safety trial of rhASM in Type B NPD patients, the highest safe initial dose was determined to be 0.6 mg/kg [24]. Due to the very high doses of rhASM used in the current study (25 mg/kg q.72 h), we examined the potential toxicity of the combination treatment by monitoring the body weights throughout the study and by examining the liver function of the mice at the end of the treatment. The weights at the start and at the end of the treatment were not significantly different (Figure S1A). In addition, no significant difference in alanine transaminase (ALT) was observed in either sorafenib or rhASM/sorafenib treated mice compared to control (Figure S1B). Two animals with high outlier values of ALT had pockets of inflammatory cells (Figure S1B) in an otherwise healthy liver, with no signs of chronic injury (Figure S1C). Of note, the long-lived rhASM/sorafenib mice ID#452 (ALT 52 U/L) and ID#443 (ALT 53 U/L) were not the outliers. Aspartate transaminase (AST, Figure S1D) and total bilirubin (Figure S1E) also were not significantly changed by the combination treatment. Together, these data suggest that the combination of rhASM (25 mg/kg q.72 h) and sorafenib (30 mg/kg q.d.) is well tolerated.

### Evaluation of rhASM/sorafenib treatment in an orthotopic model of Huh7 tumors

**Figure 5:**
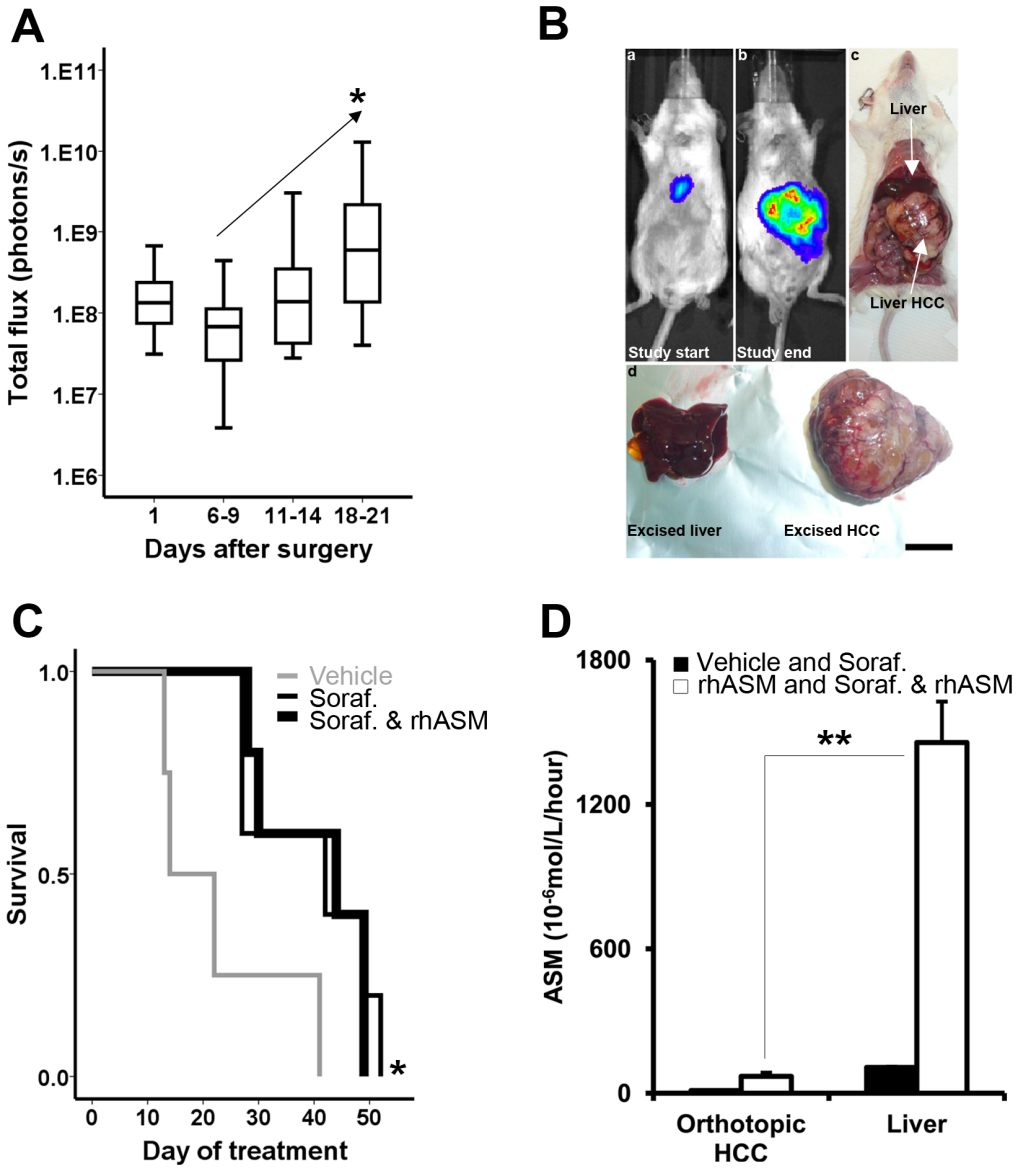
Orthotopic model of Huh7 tumors confirms the findings of rhASM/sorafenib treatment observed in subcutaneous Huh7 xenografts. (A) After surgical injection of luciferase-labeled Huh7 cells, luminescence was monitored over time. Increase in luminescence ≥6 fold baseline (day 1) was used as evidence of successful implantation of cells, initiation of tumor growth and a point of randomization of mice into treatment groups. (B) Representative images of mice at the beginning of drug treatment showing well detectable luminescence from the liver (insert a), large luminescent area corresponding to the liver Huh7 tumor at the time of sacrifice (inserts b,c). Liver and tumor were excised and separated (d) for further processing. Scale bar in insert d is 1 cm. (C) Significantly longer median survival of sorafenib treated mice (n = 5, 42 days, chi-square 4.88, df (1), p = 0.027) and combined rhASM/sorafenib treated mice (n = 5, 44 days, chi-square 4.43, df (1), p = 0.035) was observed compared to control (n = 4, 14 days). No significant difference was observed between rhASM alone (n = 4, 19 days) and control or between sorafenib and rhASM/sorafenib. (D) rhASM activity in orthotopic HCC from vehicle and sorafenib treated mice (naïve to rhASM treatment; baseline rhASM) was significantly, 6.4±1.4 fold, lower than rhASM activity in tumors from rhASM and rhASM/sorafenib treated mice (t = −3.99, df (15), p = 0.001). Similarly, baseline rhASM in the liver was significantly lower, 13.6±1.6 fold, than activity of rhASM in livers of mice receiving rhASM or rhASM/sorafenib (t = −9.07, df (14), p = 3.1· 10^−7^). Importantly, the difference between the activity of rhASM in treated mice (rhASM and rhASM/sorafenib) between orthotopic HCC and liver was much higher in the liver, 20.8±2.4 fold (t = −8.7, df (13), p = 8.8·10^−7^, **). Note: Y-axes activity (10^−6^ mol/L/hour) was measured in equal parts of 20% weight/volume tissue extracts as described in Materials and Methods.

The above positive results of rhASM/sorafenib treatment were obtained despite poor biodistribution of rhASM to the subcutaneous tumors. We therefore reasoned that the results might be improved in an orthotopic model of HCC. To establish such a model, Huh7 cells stably expressing the luciferase reporter gene were injected into the liver parenchyma of SCID/beige mice. Animals were imaged 24 hours and 1 week after surgery, and monitored every 4–5 days until a continued increase in luminescence was observed (Figure 5A). This was done to ensure that cells survived the implantation procedure and started to expand and generate tumors. All mice had detectable luminescent areas within the liver region at the beginning of the treatment, and greatly enlarged luminescence areas at the end of the study – corresponding to the tumors growing in the liver (Figure 5B).

Randomization and initiation of treatment were performed as described in Materials and Methods. Mice receiving the rhASM/sorafenib combination were started on the same dose and treatment schedule as in the subcutaneous model – 30 mg/kg sorafenib q.d. by gavage and 25 mg/kg rhASM q.72 hours i.p. Although we were not able to accurately quantify tumor size by luminescence over time (likely due to the small number of animals and kinetics of luciferin activation within tumors), there was no apparent plateau of luminescence in the treated mice. Therefore, the frequency of rhASM administration was increased (2-days-on, 1-day-off) 2 weeks into the study in an attempt to mitigate possible sub-dosing of rhASM.

The survival profiles of the treated mice were similar to those observed in the subcutaneous model, namely no significant difference between the sorafenib alone and rhASM/sorafenib combination groups (Figure 5C). Surprisingly, however, ASM activity in the healthy livers of the treated mice was several fold greater than in the orthotopic Huh7 tumors from the same animals. This is similar to the results from the subcutaneous tumors (Figure 4B), indicating that the poor biodistribution of rhASM to Huh7 tumors was independent of xenograft location.

Given the similar ASM activity profile in the two different models we next explored the expression of two receptors important in the cellular internalization of rhASM – insulin like growth factor receptor 2 (IGF2R) and mannose receptor 1 (MRC1) – in HCC. Analysis of the Oncomine database indicated that the expression of *IGF2R* is not consistently deregulated between the 4 different human HCC data sets, while expression of *MRC1* was down-regulated in 3/4 data sets (gene ranks: top 11, 2, and 5%) (Table 2).

**Table 2:** Decreased expression of *MRC1* gene in HCC.

Gene symbol:	*IGF2R MRC1*		*IGF2R MRC1*		*IGF2R MRC1*		*IGF2R MRC1*	
Oncomine set:	Mas liver		Chen liver		Wurmbach liver		Roessler Liver2	
Liver samples:	19		76		10		220	
HCC samples:	38		102	93	35		225	
Genes analyzed:	12603		10802		19574		12624	
Fold change:	−1.413	/	1.268	−2.302	/	−2.918	1.169	−2.924
T-test:	−4.203	/	3.632	−5.399	/	−6.291	3.518	−13.849
P value:	4.9E-5	NS	1.9E-4	1.3E-7	NS	7.1E-8	2.4E-4	5.3E-36
Gene rank:	1949	/	2101	1111	/	228	4761	517
Gene rank %:	Top 16%	/	Top 20%	Top 11%	/	Top 2%	Top 11%	Top 5%
mRNA in HCC:	↓	−	↑	↓	−	↓	↑	↓

No clear change in *IGF2R* expression, and significantly lower expression of *MRC1* in HCC versus liver in 3/4 human datasets (Chen Liver [18], Wurmbach liver [19], and Roessler Liver 2 [20]). NS not significant,/values not shown, ↓ under-expressed, ↑ over-expressed, − no change, Mas liver [17].

We therefore investigated the expression of *MRC1/Mrc1* in livers and orthotopic Huh7 tumors. Two different sets of PCR primers were designed to estimate the expression of human and mouse *MRC1* and *Mrc1*, respectively. The results (Table 3) revealed no detectable expression of *MRC1* in human Huh7 cells or the orthotopic Huh7 tumors. Expression was detected in healthy human liver as a positive control. The expression of mouse *Mrc1* was detectable in non-tumor liver and in the Huh7 tumors. The latter is in line with host-graft “contamination” which has been documented before [25]. Given that the C_T_ values of ∼21 were observed for both *Mrc1* and the *Srsf4* housekeeping gene in mouse liver, and ∼25 for *Mrc1* and *Srsf4* in Huh7 liver tumors, the expression of *Mrc1* in the tumors appears to be ∼10 fold lower. *Srsf4* was used as a housekeeping gene based on the stable expression during different stages of HCC development [26]. Overall, these results revealing very low *MRC1* expression in Huh7 cells/tumors were consistent with the low ASM activity observed in the xenograft tumors after treatment.

**Table 3:** No detectable *MRC1* gene expression in Huh7 orthotopic tumors in SCID mice.

Primer specificity:	Water (C_T_)	Huh7 cells (C_T_)	Human liver (C_T_)
* MRC1* human	–	–	21.65±0.13
* Mrc1* mouse	–	–	–
* SRSF4* human	–	20.14±0.01	21.97±0.08
* Srsf4* mouse	–	–	–
**Sample analyses:**	***MRC1*** (C_T_)	*Mrc1* (C_T_)	*Srsf4* (C_T_)
Non-tumor liver	**–**	21.3±0.3	21.2±0.2
Huh7 liver HCC	**–**	24.6±1.0	25.6±1.3

No detectable expression of human *MRC1* in Huh7 orthotopic xenograft tumors in mice. Mouse *Mrc1* mRNA is detectable in Huh7 tumors, albeit at ∼10 fold less than in liver. This expression profile of mannose receptor may contribute, at least in part, to hampered activity of rhASM at the dosing and administration regimen used in the present study. Data represent mean C_T_ values ± standard deviation. Tissues used in sample analyses were from rhASM/sorafenib treated mice. Standard deviation in ‘Primer specificity:’ reflects technical reproducibility. Standard deviation in ‘Sample analyses:’ also reflects variability between animals. C_T_ threshold cycle, − undetermined C_T_ during 40 qPCR cycles.

## Discussion

Changes in the membrane lipids of tumor cells, including glycosphingolipids, have been recognized for over 40 years [27]. Since then, the structural roles of sphingolipids have been expanded to include an intricate network of bioactive lipids with diverse roles in many cell processes, including cell death and survival. Today, the important roles of two of these lipids, ceramide and S1P, have been well established in cancer. The focus on ceramides and S1P for cancer therapy is well placed since maintaining a proper ceramide/S1P balance is critical to determining cell fate, and altered sphingolipid metabolism is a common feature of many cancers, leading to reduction in ceramide and/or elevation of S1P [10]. Thus, sphingolipid drugs under development are aimed at restoring this metabolic balance and/or enhancing ceramide-mediated death of tumor cells or tumor microvasculature [10], [11]. Several therapies based on either elevating pro-death ceramide or reducing pro-survival S1P are actively under investigation, including the use of ceramide analogues and inhibitors of ceramidases or sphingosine kinases [28]. Over the past decade numerous papers have elucidated the roles of ceramide, and ASM in particular, in cell signaling and the potential of modulating this pathway in cancer treatment [14], [29], [30]. Kolesnick and colleagues was the first to suggest that the lysosomal enzyme, ASM, may have a role in these processes, and demonstrated the importance of ASM-generated ceramide in the radiosensitivity of tumor cells and tumor microvasculature [31].

Here, we bring attention to a potential application of sphingolipid modulation in experimental HCC by using rhASM, which has been produced for human use and evaluated for enzyme replacement therapy in Type B NPD patients. HCC is a particularly deadly solid malignancy with a growing global incidence and mortality [32]. In part, unpromising outcomes in HCC patients are due to the pathogenesis of the disease, which includes the aberrant activation of major signaling pathways such as RAF/MEK/ERK, PI3K/AKT/mTOR, WNT/β-catenin, IGF, HGF/c-MET and angiogenesis [2]. This has led to the approval of the multikinase inhibitor, sorafenib, for HCC, albeit with very modest clinical effects. Therefore, to combat such a heterogeneous malignancy and improve outcomes in HCC, investigations into alternative/supportive drug treatments to sorafenib have been gaining momentum [6].

The rationale for using rhASM enzyme as an adjunctive therapy to sorafenib for HCC lies in the well recognized importance of sphingolipids in liver diseases [33], and the fact that systemic administration of rhASM leads to preferential uptake by the liver. While it may be worthwhile in the future to explore modifications of rhASM and/or selective delivery methods, as we have previously investigated [34], here we first focused on the potential impact of non-modified rhASM for which significant safety and regulatory approvals are in place for human use in Type B NPD patients. Such an advanced stage of characterization/approval for a biopharmaceutical lends rhASM to a potential fast-track translation to much needed immediate health benefits in unresectable HCC, pending successful proof-of-concept and follow up pre-clinical safety and efficacy studies in several different animal models of HCC.

Surprisingly, little is known about deregulation of sphingolipid metabolism in HCC. Analyses of available human data from the Oncomine database suggested to us that sphingolipid deregulation in HCC is important, and that a thorough data mining of deregulated lipid metabolism in HCC is warranted in order to provide additional clues for understanding the role of sphingolipids in HCC, and for developing effective therapeutic strategies using sphingolipid modulators. As evident from Table 1, comparing the expression of genes in HCC to normal liver in the Oncomine database revealed that two of the key enzymes involved in sphingolipid metabolism were downregulated in HCC samples - the *SMPD1* gene, which encodes ASM, and the *SGPP1* gene, which encodes S1P phosphatase. Decreased expression of ASM may suggest a lower potential of HCC cells to generate ceramide, and decreased expression of S1P phosphatase may suggest accumulation of the pro-survival lipid, S1P. Both of these events would favor cancer progression. Indeed, overexpression of S1P-generating SPHK1 was recently demonstrated in human HCC samples, and *in vitro* experiments illustrated enhanced migration and invasion of HCC cells overexpressing SPHK1 [35]. Based on the above, we chose to evaluate the use of rhASM as an adjunctive therapy to sorafenib in experimental HCC.

Previously, we demonstrated that rhASM treatment alone (1 µM) had no reproducible effect on the viability of 60 cancer cell lines encompassing leukemia, non-small cell lung, colon, CNS, melanoma, ovarian, renal, prostate and breast tumors [15], [16]. We anticipated that similar results would be observed in human hepatoma cells, of which we selected three of the most commonly used types, Huh7, Hep3B and HepG2. The specific roles of the various enzymes and lipids involved in ceramide/S1P signaling, e.g., sphingosine kinases and anti-apoptotic S1P itself, represents an under-investigated and important area of research in liver cancer. In this study we have only provided a snapshot of sphingolipid related changes in Huh7, Hep3B and HepG2 cells by analyzing three enzymes involved in sphingolipid metabolism, acid ceramidase (AC), sphingosine kinase 1 (SPHK1), and ASM. Our goal was simply to confirm the use of Huh7 cells for the xenograft studies and the evaluation of rhASM/sorafenib treatment, but in the future a more detailed and comprehensive analysis of the “sphingolipidomics” of liver cancer would be of great interest.

Based on the results of baseline activity of ASM, AC, and SPHK1 in these cells, we selected Huh7 cells for subsequent experiments since they had moderate levels of ASM and SPHK1 activity (Figure 1A). *In vitro*, we did not observe a significant effect of rhASM alone on Huh7 viability (88±4%), but combined rhASM and sorafenib treatment lowered cell viability significantly (50±4%), more than sorafenib alone (64±5%, Figure 1C). This suggested a synergistic action of the two drugs. Of note, transient acidification of media (2 hours, pH 6.5) was necessary to allow the effects of rhASM to be observed, in accordance with the optimal activity of rhASM below physiological pH 7.4 [36].

Since Huh7 cells are not very tumorigenic in the livers of BALB/C nude mice [37], to evaluate the effects of rhASM *in vivo* we first used a well-established model of Huh7 subcutaneous xenografts. High dose (25 mg/kg q.72 h) of rhASM was used to ensure that the enzyme would reach the subcutaneous tumors. While no effect of rhASM alone was observed on tumor volume or on mouse survival, the rhASM/sorafenib combination afforded a significantly lower tumor volume than sorafenib treatment alone (Figure 2). A trend towards better survival in the rhASM/sorafenib treatment group also was observed, although this did not reach statistical significance over sorafenib. We hypothesized that at least in part this observation could be attributed to the subcutaneous location of the tumor and modest biodistribution of rhASM to this site (see Figure 4 and below).

The *in vivo* efficacy of rhASM/sorafenib combination was confirmed by histological analyses of the tumor tissues, which showed higher necrosis and lower blood vessel counts than the sorafenib treated group. It is well established that ASM is particularly important in cell death of endothelial cells [14], [31], which is consistent with the effects shown here on reduced blood vessel density in the rhASM/sorafenib combination group using anti-α SMA and anti-CD34 blood vessel markers (Figure 3). A clear difference between the two analyses was the lower number of blood vessels identified in tumors from control animals by anti-αSMA staining (9±0.6) compared to the number identified by anti-CD34 staining (11.6±0.9). This apparent discrepancy is the result of different target selectivity of the individual antibodies used. Anti-CD34 recognizes endothelial cells, which are present in all blood vessels, while anti-αSMA recognizes smooth muscle actin, which is only found in vessels that have the smooth muscle layer. Given the ongoing angiogenesis in the tumor and the sequelae of events in the generation of new blood vessels, it is understandable why an antibody that recognizes endothelial cells would capture blood vessels that have not yet formed and/or do not have the muscle layer. Therefore, the greater number of blood vessel is detected in control animals using an anti-CD34 staining. Of note, in the literature one example utilizing the difference in staining of CD34 and αSMA is to improve diagnosis in astrocytoma [38], something that is beyond the scope of the present manuscript.

Importantly, both staining methods revealed almost identical values for the number of blood vessels in tumors of treated mice, which suggested that the treatments exerted an anti-angiogenic effect, and that the rhASM/sorafenib combination provided additional benefit. Overall, these results suggest that the synergism of the two drugs *in vivo*, and that the effects in this subcutaneous model of Huh7 tumors, may be largely due to reduction of blood supply rather than effects on proliferation of the tumor cells themselves. The analysis of ceramide levels in tumors, which showed no difference between the groups (data not shown), was done as an endpoint measurement at the completion of the study (up to 48 hours after the last drug injection). Since the elevation of ceramide in cells in response to rhASM is rapid and often returns to baseline within minutes, we looked at tumor necrosis and blood vessel density as surrogate markers for the biological effects observed after chronic administration (up to 43 days) of rhASM/sorafenib. Since we observed a decrease in tumor volume, increase in necrosis, and decrease in blood vessel density, we did not measure the levels of other sphingolipid metabolites such as S1P. In general, it is clear from our data that the predominant effect of rhASM combination treatment was cell death, and thus any downstream S1P that may have been generated did not prevent these rhASM/sorafenib induced changes.

To evaluate the distribution of rhASM to the subcutaneous tumors, the amount of enzyme at the end of study was examined by measuring the ASM activity (Figure 4). The ASM activity in tumors was 2 fold greater in the rhASM/sorafenib treated mice compared to vehicle or sorafenib groups, while the ASM activity in the livers was almost 40 fold greater. Previous studies have demonstrated high ASM activity in the liver following a bolus intravenous administration of rhASM [36]. Together, these data demonstrated that the distribution of rhASM to the subcutaneous tumors was modest, and likely responsible for the limited clinical effects of rhASM/sorafenib treatment observed *in vivo*.

Next, we examined the safety of high dose rhASM treatment. Manageable toxicity is particularly important in HCC patients who have cancer in addition to diseased livers (e.g., viral hepatitis, cirrhosis). In a phase I safety study of rhASM in NPD patients, the dose of rhASM did not exceed 1 mg/kg i.v., with hyperbilirubinemia detected in 1/11 patients [24]. While NPD disease is a unique situation in which the sphingomyelin load is exceedingly high, it points to the potential of rhASM related toxicity in HCC patients. The results of our current study showed that an i.p. dose of 25 mg rhASM/kg q.72 h did not produce acute toxicity, death, weight loss or significant alteration of liver function in BALB/C nude mice during up to 6 weeks of treatment in combination with sorafenib. This is consistent with the fact that i.v. doses of up to 30 mg rhASM/kg were well tolerated in larger (non-NPD) animals and primates (personal communication E.H.S.).

The rationale for using a subcutaneous HCC model was based on the fact that a) other preclinical studies of sorafenib were carried out using subcutaneous tumor xenografts [39], b) we have previously shown that rhASM is a beneficial adjunct to irradiation therapy in subcutaneous model of melanoma [14], c) recent studies showing the beneficial effects of modulating sphingolipid signaling in cancer treatment were done using subcutaneous models of liver cancer [12], [40], and d) subcutaneous models have been used for decades in assessing antitumor activity of new drugs. They are minimally invasive and effective, allowing straightforward visualization of tumor induction and quantification of tumor growth.

During the preparation of the present manuscript SCID/beige mice were used to successfully induce orthotopic liver tumors by splenic injection of Huh7 cells [22]. Indeed, SCID mice may be a better host than nude mice considering the significant differences in success of establishment of human xenografts in these two hosts [41]. We therefore modified the procedure of [22] as described in Materials and Methods, and investigated the efficacy of rhASM/sorafenib combination in the orthotopic Huh7 tumors in SCID/beige mice.

Orthotopic tumors (Figure 5 A,B) were allowed to grow until a significant impairment such as distention of abdomen, ruffled hair coat, and/or reduced/weak movement were observed, at which time the mice were sacrificed and survival data recorded. Tumor burden at the time of sacrifice (tumor weight/mouse weight) was 20.5±1%. Survival profiles of mice with orthotopic tumors was similar to that in subcutaneous model (Figure 2), namely no significant difference was observed between the sorafenib and rhASM/sorafenib groups (Figure 5C). Surprisingly, we also observed similar activity of rhASM in non-tumor liver and orthotopic HCC as in the subcutaneous model (Figure 4B). Namely, the activity of rhASM in livers of treated mice was ∼20 fold higher than in an orthotopic tumor of the same mouse (Figure 5D). The higher fold difference between liver and HCC (∼20 fold), compared to subcutaneous model (∼12 fold greater activity in liver than tumor), was likely due to higher frequency of rhASM administration in the orthotopic study (5 injections per week versus 3/week). Considering that rhASM has been developed for the treatment of NPD [42], and with continued evidence regarding the importance of ASM in preventing tumor growth and development [43], [44], we decided to examine the reason for low ASM activity in the Huh7 tumors more closely.

The rhASM used in the present study is internalized by a combination of insulin growth factor 2 receptor (IGF2R), also known as the mannose-6-phosphate receptor [45], and by the mannose receptor MRC1. We have previously shown that the uptake of rhASM in human alveolar macrophages is almost completely blocked (∼90%) by the mannose receptor ligand – mannan [46], and inhibition of uptake by mannose-6-phosphate (IGF2R ligand) is about 50% [36], [47]. It is known that the expression of *IGF2R* is monoallelic in mice, and biallelic in most humans [48]. However, *IGF2R* – a tumor suppressor in liver cancer [49], [50] – is mutated in HCC with loss of heterozygosity [51]. *MRC1*, on the other hand, is expressed in liver sinusoidal endothelial cells and liver macrophages [52] and less investigated in liver cancer.

Analysis of the Oncomine database revealed modest differences in *IGF2R* mRNA expression and inconsistent direction of the change in expression between HCC and liver, and rather low gene rank (Table 2). On the other hand, *MRC1* was significantly down-regulated in 3/4 datasets with a combined total of 306 liver and 353 human HCC samples analyzed (Table 2). We therefore examined the expression of *MRC1* in Huh7 orthotopic tumors and non-tumor mouse liver (Table 3). qPCR data showed no detectable human *MRC1* expression in the Huh7 tumors compared to the positive control (healthy human liver). Low level expression of mouse *Mrc1* expression in the orthotopic tumors was likely due to host-graft contamination inherent to xenograft models in mice [25]. The lack of detectable *MRC1* expression in Huh7 xenografts could be due to a known decrease in the number of Kupffer cells in HCC, particularly in larger and poorly differentiated tumors [53], [54], [55]. *MCR1* is expressed at high levels in this cell type. Taken together, these data suggest that the efficacy of rhASM treatment in HCC may be hampered by tumor pathology and the pharmacokinetics of the rhASM, namely the high uptake by non-tumor liver and low uptake by HCC cells.

In summary, here we demonstrate the potential of rhASM as an adjuvant to sorafenib treatment of experimental liver cancer. The reduced tumor volume in the combination treatment using the subcutaneous model of HCC, as well as the trend towards extended survival, appears to be largely due to a synergistic action of rhASM and sorafenib on reduction of blood vessels in tumors, resulting in an increase in necrosis. The ∼12 times higher ASM activity in healthy livers than in subcutaneous tumors highlighted the hepatotropic nature of rhASM during chronic administration. Moreover, the higher activity of rhASM in non-tumor liver versus tumors in the orthotopic model (∼20 fold higher) further suggested that healthy liver may be retaining the majority of injected rhASM, preventing it from accumulating in the tumors. The tumor cells themselves also do not express one of the two key receptors involved in the cellular internalization of rhASM – MRC1 – which also likely contributes to the diminished capacity of rhASM to reach HCC tumors *in vivo*.

The present study highlights the need to closer examine the pharmacokinetics of rhASM in order to design a more tumor-selective/targeted enzyme and lessen the capture by non-tumor liver. Examples of potential approaches to enhance the anti-tumor effect of rhASM would include modifications of sugars which can alter receptor internalization of the enzyme as in Gaucher's disease [42], or additionally, use of nano-carriers targeted to e.g., tumor endothelial cells and loaded with the rhASM enzyme [47]. The latter should take into account the potential caveats of developing multi-functional carriers, most importantly the preparation and purification of such nano-particles, the stability following the introduction of targeting moieties, and lack of over-expression of a specific target receptor in a heterogeneous malignancy such as HCC [56], [57]. Lastly, in addition to the approaches discussed above and given the complexity of molecular aberrancies in unresectable HCC, combining strategies to increase ceramide levels (e.g., rhASM) with those that prevent ceramide degradation (e.g., ceramidase inhibitors) and/or production of S1P (e.g., SPHK1 inhibitors) deserve additional consideration.

## Materials and Methods

### Measurement of baseline enzymatic activities in hepatoma cells

Human Huh7, HepG2, and Hep3B cells were cultured in Dulbecco's modified Eagle's medium (DMEM) supplemented with 1% penicillin (100 U/mL), 1% streptomycin (100 µg/mL) and 10% fetal bovine serum. All tissue culture flasks and dishes were from BD Biosciences (Bedford, MA), and media was from GIBCO (Invitrogen, Carlsbad, CA). Enzymatic activity of AC, SPHK1, and ASM was determined using high performance liquid chromatography as described previously [36], [58].

### In vitro rhASM effect on cell viability

Huh7 cells were seeded in 96 well plates (5,000/well) and incubated overnight. They were treated the next morning for 2 hours with rhASM (500 µg/mL) in a medium acidified with 2-N-morpholino ethane sulfonic acid (M-8250, Sigma), with or without addition of sorafenib (3 µM). Controls included cells treated with acidified medium (pH 6.5), DMSO (<0.001%) and rhASM vehicle (0.1 M D,L-methionine, 5% sucrose, 20 mM sodium phosphate, 0.1 mM EDTA). The media was changed after 2 hours to non-acidified medium containing the same control and enzyme/drug treatments. Viability was then examined after 46 hours by an MTT assay. Data are expressed as % of control.

### In vivo effect of combined rhASM/sorafenib treatment in subcutaneous Huh7 tumors

Animal studies were performed following approval by the Institutional Animal Care and Use Committee. Five million Huh7 cells/100 µL of phosphate buffered saline were aspirated into 1 mL syringes (BD 309602) and injected subcutaneously into female BALB/C nude mice (regio costalis/hypochondriaca) using a 25^5/8^ gauge needle (BD 305122). Tumor length and width was measured in mm using a manual caliper, and tumor volume estimated as length•width^2^•0.4. The mice developed tumors measuring 100–150 mm^3^ as early as 18 days after the injection of cells, and inclusion of mice in the study was closed two weeks thereafter. Mice were randomized to vehicle, rhASM, sorafenib/rhASM combination, and sorafenib treatment groups. Sorafenib (LC labs) was sonicated in chremophore/ethanol/water (0.125∶0.125∶0.75) using a water bath, and administered by gavage 30 mg/kg q.d. rhASM was administered by i.p. injection 25 mg/kg q.72 h. Vehicle treatments included chremophore/ethanol/water gavage and rhASM buffer injection (0.1 M D,L-methionine, 5% sucrose, 20 mM sodium phosphate, 0.1 mM EDTA). Mice were sacrificed when the tumor volume reached 1,000 mm^3^ except for 2 mice in the combination arm whose tumors measured less than 300 mm^3^ after >40 days of treatment, and which were sacrificed at day 43. Tumors, livers, and sera were collected at the time of sacrifice and frozen on dry ice or fixed in 10% paraformaldehyde in phosphate buffered saline.

### In vivo effect of combined rhASM/sorafenib treatment in orthotopic Huh7 tumors

Animal studies were performed following approval by the Institutional Animal Care and Use Committee. Huh7 cells were stably transduced using Cignal™ Lenti Positive Control (luciferase) lentiviral particles (CLS- PCL-1; SA Biosciences) following the manufacturer's instructions. Luminescence was assayed in cells stably expressing luciferase using a Luciferase Assay Kit (E1501; Promega) and a Veritas Microplate Luminometer (Promega). Five million luciferase expressing Huh7 cells/20 µL of phosphate buffered saline was aspirated into ½ mL insulin syringe (BD 329461) with a permanently attached 28^½^ gauge needle. Mice were anesthetized with isofluorane, and laparatomy performed to expose the left liver lobe. Cells were then injected into liver parenchyma. One day after surgery the baseline luminescence was recorded using an IVIS system. Mice were then imaged every 3–4 days and randomized to treatment upon registering a continued increase in luminescence (≥6 fold baseline). Mice were randomized to vehicle (n = 4), rhASM (n = 4), rhASM/sorafenib (n = 5), or sorafenib (n = 5) treatment groups. Sorafenib (LC labs) was sonicated in chremophore/ethanol/water (0.125∶0.125∶0.75) using a water bath, and administered by gavage 30 mg/kg q.d. rhASM was administered by i.p. injection 25 mg/kg q.72 h. Two weeks into treatment the frequency of rhASM administration was increased to a two-days-on/one-day-off schedule. Vehicle treatments included chremophore/ethanol/water gavage and rhASM buffer injection (0.1 M D,L-methionine, 5% sucrose, 20 mM sodium phosphate, 0.1 mM EDTA). Mice health status and tumor burden were assessed daily. Mice were sacrificed when tumor growth (e.g., considerable distention of abdomen), grooming (e.g., ruffled hair coat) and/or movement (e.g., hunched posture, reluctance to move) were observed. Tumors, livers, and sera were collected at the time of sacrifice and frozen on dry ice or fixed in 10% paraformaldehyde in phosphate buffered saline.

### Immunohistochemistry

Paraformaldehyde fixed paraffin embedded tissues were sliced into 5 µm thick sections and processed as follows. Slides were baked at 59°C for 30 minutes followed by 5 minute washes in xylene. Rehydration was completed by 5 minute rinses in 100%, 95%, 85%, 70% ethanol followed by a rinse in water. Heat antigen unmasking was done in sodium citrate (pH 6) using a microwave (3×5minutes at high power). Endogenous peroxidases were blocked (TR-015-HD kit), followed by incubation in 5% albumin in PBS (1 hour at room temperature). Five % goat serum (S-1000, Vector Laboratories Inc.) in PBS was used as a blocking agent prior to blood vessel staining. Ki67 (H-300, Santa Cruz), αSMA (ab5694, Abcam), and CD34 (NB600-1071SS, Novus Biologicals) antibodies were used (1∶150, 1∶400, 1∶250) to assess the proliferation index and blood vessel density, respectively. Primary antibody incubations in 5% FBS and 5% BSA in PBS (Ki67) or 5% goat serum in PBS (αSMA, CD34) were carried out at room temperature for 1 hour. The remaining procedures were performed as suggested by the manufacturer (TR-015-HD kit, Thermo Scientific), and slides dehydrated by reversing the order of solvent incubations (water ethanol xylene) and coverslipped using permount. Hematoxylin and eosin staining was done by the Mount Sinai Histology Service Shared Resource facility. Proliferation (Ki67) was quantified by counting Ki67 positive and negative nuclei and expressing the result as a percentage of positive to total (positive + negative) nuclei. TIFF images from three random fields were examined per slide using the GIMP 2.6.11 software. Each image, 2048×2048 pixels, was overlaid with a grid of 500×500 pixels and cell nuclei counted in five 500×500 squares. Necrosis in the tumor sections was visually assessed by a liver cancer pathologist (I.M.F.), and independently by R.S. using the Image J software (percent of necrotic area compared to the total area of the tissue section). Blood vessels were counted in up to 10 fields per slide, 200× magnification, for both the αSMA and CD34 markers, and an average calculated for each sample. The results were expressed as a mean number of blood vessels per 200× field for each treatment group.

### ASM enzymatic activity in tumors

Tissue extracts (20% weight/volume) were prepared using Igepal (I3021, Sigma) and protease (78420, Thermo Scientific) and phosphatase inhibitors (1862209, Thermo Scientific) lysis buffer and a hand-held tissue homogenizer (Tissue Tearor 985370). The extracts were frozen at −80°C until the analyses. Equal parts of tissue homogenate and reaction buffer (200 µM Bodipy-C12-sphingomyelin; 0.2 M sodium acetate buffer pH 5.0, 0.2 mM ZnCl_2_, and 0.2% Igepal CA-630) were incubated at 37°C for 30 minutes. The reactions were stopped by dilution of the samples in absolute ethanol (1∶40), and activities analyzed as described [59].

### Liver function tests

Mice were exanguinated by orbital venipuncture and blood collected in serum separator tubes (BD 365956), centrifuged at 4° C, and serum frozen at −80° C. Aliquots of sera (100 µL) were brought up to a total volume of 200 µL with 5% BSA and submitted for analyses of ALT, AST and total bilirubin to the Mount Sinai Center for Clinical Laboratories.

### Quantitative real-time PCR (qPCR)

The RNeasy® Mini Kit (74104, Qiagen) was used to extract cellular RNA following the manufacturer's instructions and included a genomic DNA digestion step (RNase-free DNase Set 79254, Qiagen). Human total liver RNA was purchased from Clontech as a positive control (636531). One and a half µg of RNA was reverse transcribed using the High Capacity cDNA Reverse Transcription Kit (4368814, Applied Biosystems). cDNA was diluted 1∶15 prior to running the qPCR. Primers used for SYBR® green I detection (iQ™ SYBR® Green Supermix (1708885, Bio-Rad)) were as follows: *MRC1* (Forward: CGT CTT CTG GGT TTT GGA GT, Reverse: GGG TCC ATC TTC CTT GTG TC), *Mrc1* (Forward: GCG TTG CAC ATA CCT CAA GA Reverse: GCT AAA TGA TCG CAT GCT CA), *SRSF4* (Forward: TGG AAC TGA AGT CAA TGG GAG, Reverse: CTG CTC TTA CGG GAA TGT CTG), *Srsf4* (Forward: GCT CTC GAA GCA GAC ATT CC, Reverse: AGT GGG AAC CTG ACC TGG AT). The annealing temperature was optimized using a S1000™ Thermal Cycler (Bio-Rad), and qPCR was run using the 7900HT Applied Biosystems Real-Time PCR System (Stage 1: 95°C 2:00 (1 cycle); Stage 2: 95°C 0:20, 57.7°C 0:15, 72°C 0:10 (40 cycles); Stage 3: dissociation stage). Data were analyzed using SDS 2.2.1 software.

### Statistics

All analyses were done using the IBM SPSS Statistics 19 software. Unless otherwise stated multiple group comparisons were analyzed by one-way ANOVA, followed by 2-sided Dunnett's or Tukey's post-hoc test. Pair wise comparisons were done using independent 2-sided student's T-test. P values less than 0.05 were considered significant.

## Supporting Information

Figure S1 **Normal liver function tests in rhASM/sorafenib co-treated mice.** (A) Weights at the end of treatment were not different between vehicle and drug treatment groups (ANOVA, df (2,30), F = 0.51, p = 0.608). Within groups, no difference between the start and end weights was detected in vehicle (t = −1.05, df (16), p = 0.308), sorafenib (t = 1.08, df (18), p = 0.294), or rhASM/sorafenib (t = 0.525, df (26), p = 0.604) treated mice. (B) Measurements of ALT in mouse sera showed no significant difference between the groups (ANOVA, df (2,30), F = 1.689, p = 0.202). Two outliers (*) in the rhASM/sorafenib group had elevated ALT values. Histological examination of livers from these two mice (C) revealed pockets of inflammatory cells (dotted arrow) in an otherwise normal tissue with healthy hepatocytes (arrow). Further analyses of AST (D) showed no significant changes (ANOVA, df (2,30), F = 1.949, p = 0.160). (E) Total bilirubin in the groups was normal with most values being below the lower limit of detection (<0.1 mg/dL).
(TIF)
