# Supplementary figures and images for: Recombinant Human Acid Sphingomyelinase as an Adjuvant to Sorafenib Treatment of Experimental Liver Cancer
Source: PLoS One. 2013 May 28;8(5):e65620. doi: 10.1371/journal.pone.0065620 (PMC3665770; doi:10.1371/journal.pone.0065620)

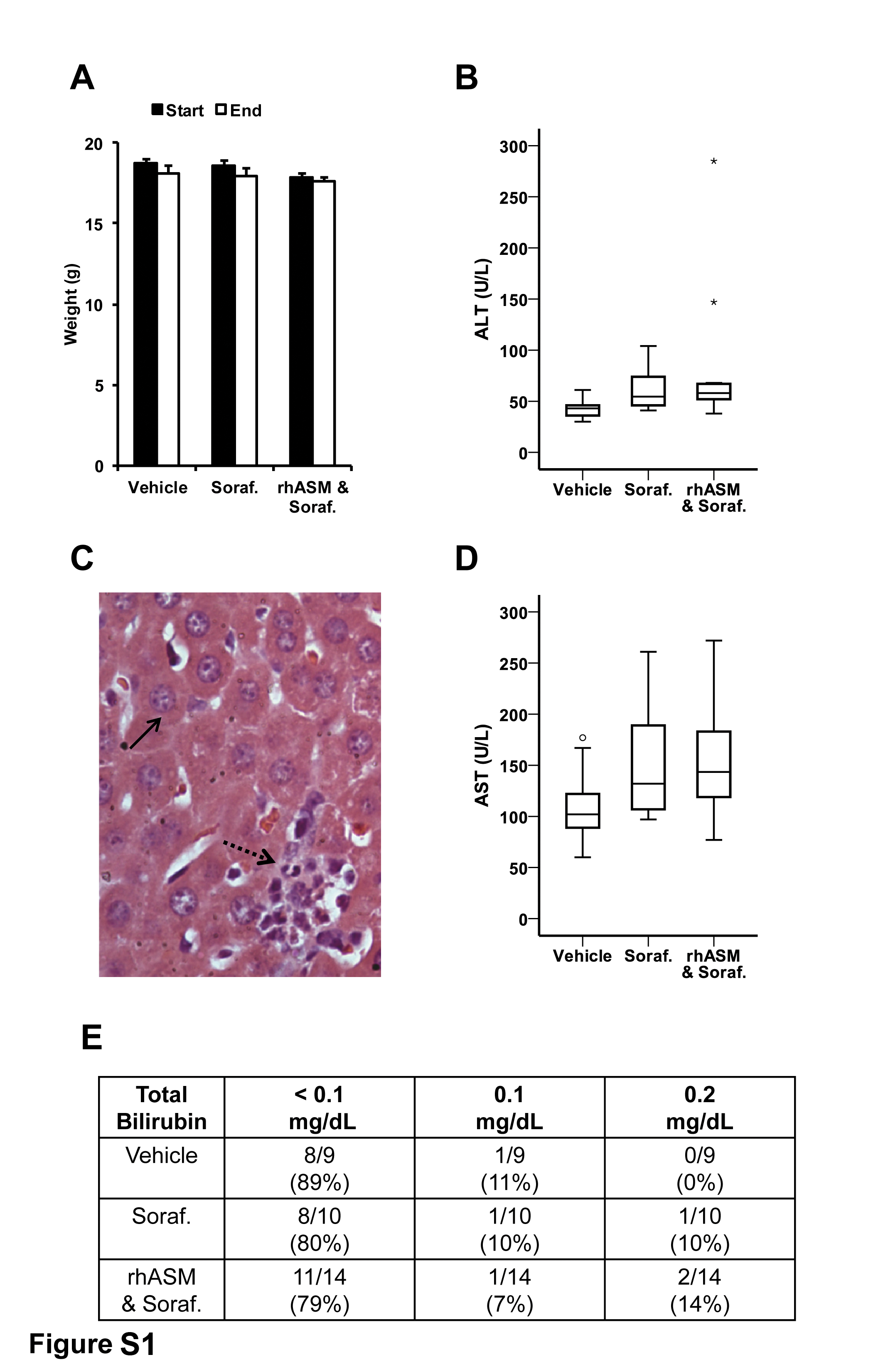

Supplement: Figure S1 — Normal liver function tests in rhASM/sorafenib co-treated mice. (A) Weights at the end of treatment were not different between vehicle and drug treatment groups (ANOVA, df (2,30), F = 0.51, p = 0.608). Within groups, no difference between the start and end weights was detected in vehicle (t = −1.05, df (16), p = 0.308), sorafenib (t = 1.08, df (18), p = 0.294), or rhASM/sorafenib (t = 0.525, df (26), p = 0.604) treated mice. (B) Measurements of ALT in mouse sera showed no significant difference between the groups (ANOVA, df (2,30), F = 1.689, p = 0.202). Two outliers (*) in the rhASM/sorafenib group had elevated ALT values. Histological examination of livers from these two mice (C) revealed pockets of inflammatory cells (dotted arrow) in an otherwise normal tissue with healthy hepatocytes (arrow). Further analyses of AST (D) showed no significant changes (ANOVA, df (2,30), F = 1.949, p = 0.160). (E) Total bilirubin in the groups was normal with most values being below the lower limit of detection (<0.1 mg/dL). (TIF) [file pone.0065620.s001.tif]
